# Supplementary material for: Are carbon emissions trading and green financial instruments synergistic? -Comprehensive quantitative research based on content analysis
Source: PLoS One. 2024 Mar 7;19(3):e0298601. doi: 10.1371/journal.pone.0298601 (PMC10919601; doi:10.1371/journal.pone.0298601)
Supplement: S1 Data — (ZIP) [file pone.0298601.s001.zip › Data/地级市Z_F工作报告.zip!/╝¬┴╓/░╫╔╜╩╨/└┤╫╘╣1⁄2╓┌║┼í╛┬φ┐╦╩2╛▌═°í┐_2012.docx]

各位代表：

　　我代表白山市人民政府，向大会作政府工作报告，请予审议。并请市政协委员和列席人员提出意见。

　　一、真抓实干，合力攻坚，2011年全市经济社会发展实现新突破

　　2011年，在省委、省政府和市委的正确领导下，在市人大、市政协的监督和支持下，我们深入贯彻落实科学发展观，坚持扩投资保增长、调结构上水平、抓开放增活力、惠民生促和谐，圆满完成了市六届人大四次会议确定的各项任务，实现了“十二五”良好开局。在中国社科院发布的中国城市竞争力蓝皮书报告中，我市综合增长竞争力在全国294个城市中位列第三。全市经济发展、民生改善、政治安定、社会稳定，步入了速度快、质量好、效益优、人民群众幸福指数明显提升的新阶段。

　　(一)坚持转型促发展，综合经济实力实现“五个突破”

　　预计全年实现地区生产总值545亿元，增长19%；全口径财政收入55亿元、地方级财政收入34亿元，分别增长27%和35%；财政支出120亿元，增长20%；城镇居民人均可支配收入、农民人均纯收入达到18000元、7300元，分别增长12%和19%。

　　——— 投资带动转型实现新突破。预计全年固定资产投资实现430亿元，增长34%。注重优化投资结构，现代服务业和新兴产业投资比重分别达到46.5%、21.3%。开工建设3000万元以上项目468个，其中亿元以上项目225个。484个转型项目加快推进，投资比重占固投总额的43%。长白山国际旅游度假区累计投资105亿元、年内投资55亿元，行政及商务会议中心等基础设施交付使用，43条雪道、6座星级酒店、温泉中心和民营经济论坛会址初具规模；总投资 30亿元的华能煤矸石发电项目顺利竣工；琦祥纸业20万吨包装用纸综合扩能改造投资3.5亿元，完成主要设备订购和部分厂房建设；10万吨二氧化硅、4万吨硅藻土助滤剂、晋吉锂电池和太阳能单晶硅切片项目开工；万达1000万吨矿泉水、山水集团400万吨水泥、浙江荣事基业长白山高档家具、武汉凯迪综合性生物质能源项目成功签约。《白山市资源枯竭城市转型规划》通过国家评审，镁工业园等7个经济转型项目挤进国家盘子。全市纳入长白山林区生态保护与经济转型规划扎实推进，浑江区列入全国首批矿产资源综合利用示范基地，长白山地质数据集成开发项目试点启动，餐厨废弃物资源化利用和无害化处理项目列为国家试点工程。

　　——— 工业提速升级实现新突破。预计全市工业总产值突破1000亿元，增长40%以上；实现规模以上工业增加值280亿元，增长30%；实现净利润25亿元，增长15.4%；新增规模以上企业30户，达到393户，提前一年实现工业产业跃升目标。市直工业转型加快，板石矿业120万吨氧化球团、修正药业熊胆开明片等一批转型升级项目全面启动，市直工业总产值实现116亿元，增长38.7%。技改投入力度加大，完成技改投资130亿元，增长35%，实现新产品产值72亿元，增长37.7%。中小企业腾飞成效明显，民营经济主营业务收入870亿元，增长53.4%。企业家信心指数比2008年提升52.6个点，创历史新高。节能减排效果显著，单位GDP综合能耗降低3.5%，化学需氧量、二氧化硫、氮氧化物、氨氮削减量分别完成省定指标的 111%、258%、105%、115%，被省政府授予公共节能优秀先进市。金融保障能力进一步增强，全市金融机构本外币各项存款余额增长17.7%，各项贷款增长14.2%，增幅均列全省第一。新组建两家民营担保机构、6家小额贷款公司，成功组织5次银企对接活动，企业和项目获得11.4亿元信贷支持。振东煤业列为全省首批发行集合债券企业，东锋镁业向国家证监会提交上市申请材料。上级扶持力度加大，全市争取沉陷区补充工程、浑江干流堤防维修加固、鸭绿江界河治理等国家、省专项资金65亿元。

　　——— 农业转型发展实现新突破。预计全年实现农林牧渔业总产值84亿元，增长18%。全面推进退粮进特战略，新增经济作物16万亩、蔬菜两万亩，新建标准棚膜3400亩，粮经菜比例由7：2：1调整到6：3：1。新增农产品加工企业60户达到580户，新发展省级龙头企业5户达到38户、市级龙头企业26户达到126户。启动建设了临江香菇、长白绿色人参国家级农业标准化示范区。成功引进日本津村、南京同仁堂、山东绿叶集团等一批人参合作开发项目，35家企业和77个品种批准为人参药食同源试点单位和试点品种，分别占全省的46%和30%，18个新产品投放市场。建成25个特种野猪规模养殖基地，饲养量达到15.1万头。百村示范、村村提升工程投资8.6亿元，开工建设430个新农村项目，新建20个示范村和20个整体推进村，125个村完成“四清四改”。改造农村泥草房667户。新建122处农村安全饮水工程，解决了5.92万农村人口和1万名农村学校师生安全饮水问题。森林林政管理形成管控有力的“防火墙”，实现连续32年无重大森林火灾。

　　——— 第三产业接续实现新突破。成功举办长白山鸭绿江文化旅游节、松花石博览会等节庆活动，松花石、长白玉、人参、蓝莓等旅游商品开发档次有新提升，江源松花石(砚)获国家地理标志产品保护。新评定三星级宾馆3家，新增旅行社5家，望天鹅风景区确定为首家省级旅游业标准化试点。预计全年完成旅游总收入39.2亿元，旅游人数416万人次，分别增长28%和20%。文化创意产业初步形成，各类文化服务、文化产品研发机构发展到300余家，成立长白山演艺有限公司，我市成为东北最大的松花石(砚)集散地，拥有省级标准制定权。坚持搞活商贸流通，完成合兴大厦改造和星泰农贸市场扩建。靖宇物流园区、长白山特产城、欧亚商场等一批商贸流通项目加快推进。“万村千乡市场工程”连锁农家店突破1200户，配送中心达到6个。预计全年社会消费品零售总额完成168亿元，增长20%。

　　——— 对外开放引资实现新突破。预计全年引进市外资金363亿元，增长44.6%，其中实际利用外资1.5亿美元，增长15%。以商招商成效明显，中国建材、广泽集团等一批战略投资者落户白山。会展招商成果丰硕，成功承办首届中国民营经济发展(长白山)研讨会，积极参加第七届东博会，签约投资合作项目37个，合同利用外资264.3亿元。招商引资环境进一步优化，被中国城市发展研究院等6家知名机构评为2011年度最佳投资城市。外经外贸快速增长，新增外贸进出口经营权企业30户，丽美坚木业成为我市首家参与境外股权收购企业。预计全年实现外贸进出口总额2.7亿美元，增长22.7%。

　　(二)坚持惠民促和谐，人民生活水平实现“三个提升”

　　——— 城乡建设品位进一步提升。编制完成“十二五”建设规划和中心城区控制性详规。市中心区“六大工程”投资21.2亿元，80个城建项目开工建设。向江大桥、浑江大桥和宝兴街、民中街等9条街路竣工，改造人行步道4万平方米。新建人防工程8728平方米。开工建设煤矿棚户区23.7万平方米、城市棚户区107.7万平方米、廉租房7.2万平方米。碱厂沟河、月牙河综合治理拆迁1750户。总投资9.18亿元的沉陷区补充工程开工17.6万平方米。投资1.26亿元浑江干流左岸堤防10公里维修加固全面竣工，达到50年一遇防洪标准，并成为城市标志性文化休闲景观。曲家营水库等水利水毁工程全部完工。“暖房子”工程列入国家级重点示范市，投资26.2亿元，新改建热源9处，改造供热管网192公里，撤并小锅炉96座，既有居住建筑节能和供热计量改造426万平方米，室内温度提高3至5℃，真正体现了暖房子、暖民心。城市人居环境明显改善，市中心区占道经营和环境脏乱差等问题有效缓解，顺利通过省级卫生城复查验收。松花江出境水质保持国家2类水体标准，鸭绿江、浑江干流达到国家3类水体标准，市中心区空气优良级别天数达到325天。交通建设投资37亿元，公路总里程达到6425公里，448个行政村通沥青(水泥)路，村屯覆盖率达89.2%。营松高速公路辉南至靖宇段通车，靖宇至松江河段进展顺利，鹤大高速公路工可研通过专家评审，白山至长春高速公路进入工可研批复阶段，松江河至长白高速公路编制完成预可研候审。交通水毁工程投资1.98亿元，修复公路199公里、桥梁59座。我市被评为全省交通发展先进市。生态城镇化建设加快，抚松新城投资10亿元，拆迁居民3100户，新建安置区81万平方米，回迁1400户21万平方米。松江镇、兴参镇二道沟村等一批村镇通过国家级生态乡(镇)、生态村专家组验收。全市城镇化率达到68.6%，提升0.4个百分点。

　　——— 社会事业发展水平进一步提升。切实落实义务教育政策，为全市各类学生补贴、减免费用5088万元。教育教学质量全面提高，平安高考工作走在全省前列。全市所有乡镇建有综合性文化站，60%村建成文化室，新建农家书屋80个，形成覆盖市、县、乡(镇)、村四级公益文化工作网络。网络双向化改造4万余户。松花石文化城二期开工建设，人参、矿泉水、奇石等文化软实力明显提升。新增市级文物保护单位12个、非物质文化遗产25项。体育事业发展加快，全国越野滑雪冠军赛获2银1铜，全省乡村健身大擂台比赛荣获第三名。继续保持低生育水平，人口出生率控制在4.5‰以内。质量兴市战略扎实推进，全市中国驰名商标达到7件，施慧达荣获中国专利优秀奖，泉阳泉行政认定为中国驰名商标。新增吉林名牌产品15个，施慧达、露水河、白山方大、地王、合兴5件商标被评为全省最受信赖、最具影响力、最具发展前景商标。

　　——— 民生保障能力进一步提升。全市新增财力的80%以上用于民生，提高7.1个百分点。医药卫生体制改革稳步推进，新型农村合作医疗参合人数38.62万人，参合率达到98.95%，财政补助资金由年人均150元提高到200元。全市公办基层医疗卫生机构实行基本药物零差率销售和网上集中采购，基本药物价格比国家零售指导价下降46%。社会保障工作稳步推进，城镇新增就业5万人，完成省定指标的127%。农村劳动力转移就业新增2.1万人，完成省定指标的112.5%。城镇登记失业率控制在3.7%以内。全市五大险种累计参保达到185.4万人次，为17.43万城镇和农村低保对象发放低保金3.8亿元。办理“五七”家属工2.9万人，发放养老金9680万元。14个农村社区服务中心投入使用。年初确定的27件民生实事有14件圆满完成、11件超额完成，两件由于调整规划设计等原因没有完成，将在明年解决。加大煤矿安监力度，依法关闭 12户煤矿。信访形势保持稳定，国家“两会”和建党90周年等特殊时期，进京非正常访实现零登记，接待群众单访人次下降43.4%、集体访人次下降16.9%。治安形势保持平稳，“清网行动”成果全省第一，撤网率达95.2%，高于全省平均水平7.3个百分点。全面启动夜治安大巡控，命案侦破率100%。民族宗教工作得到加强，军政军民更加团结，“兴边富民”行动扎实推进，白山市第二次“全国双拥模范城”通过省验收，并进入国家级命名行列。

　　(三)坚持规范促高效，政府自身建设实现“三个加强”

　　——— 社会管理创新明显加强。积极探索创新管理，加强财政审核、经济审计、项目管理等方面调研，规范出台市本级政府性债务管理、环境整治、绿化美化、物业管理、改制企业运行等方面文件，建立统计联席会议制度，做到社会管理关口前移。先后与省科技厅、旅游局、工信厅建立合作促进机制，成立了施慧达研究院和全省镁产业技术创新战略联盟。市沉陷区治理办公室、城市综合执法支队恢复到住建局，市商务局外商投资和开发区管理职能划转经合局，成立市食品安全委员会办公室，进一步理顺了部门管理职能。人才保障机制进一步完善，落实教育编制68名、医疗卫生编制71名。59个乡镇(街)建立农村集体“三资”代理和便民服务中心，初步形成了便捷、透明、高效的社会管理服务平台。白山市“五五”普法受到国家表彰，浑江区、市工商局、市综治办被评为全国法制宣传教育工作先进单位。

　　——— 行政审批效能明显加强。行政电子监察系统实现省、市、县三级联网，行政许可提前办结率由36%提高到99.3%，行政审批承诺时限整体压缩 83.1%，审批件提前办结率达到97.9%，限时办结件平均办结时限1.25天。暂停减少行政审批项目 203项，是全省行政审批事项最少的市(州)。

　　——— 绩效考评工作明显加强。深入实施政府绩效评估，率先在全省将绩效评估范围扩大到6个县(市)区政府和36个中省直单位，并在全省作典型发言。市政府高度重视人大代表建议和政协委员提案，全年高质量办理125件，面复率、办结率均达到 100%，代表、委员基本满意。

　　一年来，地震、气象、外事、民兵预备役、老龄、妇女儿童、残疾人等工作都为全市经济社会发展做出了积极贡献。

　　各位代表，即将过去的2011年，是白山经济社会精彩纷呈喜获丰收的一年。这是省委、省政府和市委统揽全局、正确领导的结果；是市人大和市政协有效监督、大力支持的结果；也是全市上下万众一心、合力攻坚的结果。在此，我代表市政府，向全市各族人民，向驻白山人民解放军指战员、武警部队官兵、公安司法干警、中省属企事业单位，向各民主党派、工商联、人民团体、无党派人士、离退休老同志，向关心和支持白山发展的各界朋友，致以崇高的敬意和衷心的感谢！

　　在看到成绩的同时，我们也清醒地认识到，前进中还存在一些困难和问题，主要是：经济总量小，增长主要依赖投资，消费和出口拉动力不强；发展方式相对落后，产业升级转型较慢，接续替代产业发展不快；公共安全和灾害事故隐患较多，全社会安全防范和自救能力较弱，突发事件应急处理能力需进一步增强；保障和改善民生任务较重，部分低收入群体生活条件亟待改善；少数干部责任意识、服务意识、工作能力有待加强，发展环境还需进一步优化等。对此，我们将采取有效措施，认真加以解决。

　　二、凝心聚力，奋力拼搏，全力推进2012年经济社会更好更快发展

　　明年是实现白山快速崛起的关键之年。世界经济复苏不稳定性不确定性上升，国内通胀压力尤存，省内各地竞争更加激烈；国家明确经济社会发展稳中求进工作总基调，继续实施积极的财政政策和稳健的货币政策；辽宁沿海经济带和长吉图开发开放先导区蓬勃发展，东北东部区域合作不断深化，我市的支点和纽带地位更加凸显。纵观国际国内环境，机遇大于挑战、动力强于压力。为此，我们一定要切实增强紧迫感和责任感，抢抓发展机遇、勇于迎接挑战、激发实干动力，努力推进经济大提速、产业大转型、社会大发展、民生大改善。

　　明年政府工作的总体要求是：认真贯彻党的十七大和十七届六中全会精神，以邓小平理论和“三个代表”重要思想为指导，深入贯彻落实科学发展观，按照“三化”统筹、“三动”战略部署和即将召开的市六次党代会要求，坚持生态立市、产业强市，以保持经济平稳快速发展为首要任务，以推动经济发展方式转变和经济结构调整为主攻方向，做大做强支柱产业，发展壮大特色产业，大力培育新兴产业，不断提升对外开放水平和自主创新能力，全力推进项目建设、招商引资，全面发展社会各项事业，加快迈向全省“较发达城市”进程，努力打造全国最平安城市、最稳定边境地区，建设富裕开放、文明和谐的新白山。

　　综合各方面因素，全市经济社会发展主要预期目标是：地区生产总值增长23%；全社会固定资产投资增长25%；全口径财政收入、地方级财政收入均增长18%；城镇居民人均可支配收入、农民人均纯收入分别增长10%和17%；城镇登记失业率控制在4.6%以内。

　　为实现上述目标，必须围绕“一条主线”，突出“五项重点”，强化“三个保障”，确保2012年各项目标任务圆满完成。

　　(一)围绕转型主线，致力于投资拉动和项目建设，着力转变经济发展方式。坚持把产业结构优化升级作为转变发展方式的主攻方向，把项目建设作为加快转型的第一抓手，通过上项目实现扩总量、调结构、促转型，着力增强可持续发展能力。全力实施“ 456”工程。

　　一是加快结构调整，高质量谋划和推进 400个转型项目。紧紧围绕经济转型，以清洁能源、林木精深加工、矿产新材料、现代医药、绿色食品为重点领域，谋划400个接续和替代型产业项目。重点推进赤松核电站等项目，加快厄贝沙坦、褐煤提质、玄武岩拉丝项目建设，大力推进煤矸石、粉煤灰、低品位硅藻土、木材剩余物等一批循环经济项目。二是力促投产达效，高强度推进 500个重点项目。全年固定资产投资 538亿元，计划安排 3000万元以上重点项目500个，完成投资 400亿元。全力以赴推进长白山国际旅游度假区、琦祥纸业综合扩能改造、120万吨氧化球团、晋吉锂电池等 200个续建项目；千方百计落实开工条件，加快万达 1000万吨矿泉水、山水集团 400万吨水泥等300个新项目开工建设。三是完善推进机制，高规格包保 60个重大项目。继续实行项目领导包保责任制，确定市级领导包保 30个重大项目、县(市)区及市直相关部门领导包保30个大项目。积极引入民间资本，加大矿产资源整装勘查开发力度，增强项目发展后劲。不断完善项目推进和管理机制，对全市投资 3000万元以上项目建档立案，严格实施项目法人责任制、施工合同制、招投标制和监理制，加大重大项目组织推进力度，做到建成一个、达标一个、验收一个。

　　(二)突出提速增效，致力于结构优化和产能扩充，着力推进新型工业化进程。坚持抓投资扩产能、抓项目促调整、抓要素保运行，全面提升工业发展水平。规模以上工业增加值增长25%；实现净利润30亿元；新入统规模以上工业企业 20户；新产品产值率达到7.5%。

　　一是努力壮大工业经济总量。坚持引增量、活存量。依托八宝循环经济工业园推进硅镁铁铝等系列产业集群，谋划推进年产20万吨电解铝、5万吨镁合金、40万件稀土镁合金、2.5亿平方米纸包装箱等68个亿元以上重点工业项目。全年工业固定资产投资增长25%，其中技改投资增长33%。加快改制企业扩能增效，全力支持市直 24户规模以上企业快速扩张。实施民营经济新一轮腾飞计划，大力推动民营经济上层次、扩规模、增效益，主营业务收入增长24%，实缴税金增长23%。二是着力破解工业发展瓶颈。坚持抓要素、促保障。完善各级领导联系包扶重点企业和部门帮办制度，支持各商业银行开展金融创新，积极向上争取贷款规模，发挥好担保资金作用，完善企业和个人征信体系，积极推进“诚信白山”建设，优化银企保共赢的金融生态环境。加快推进东锋镁业、琦祥纸业上市进程，力争 1户企业挂牌上市。加强企业技术创新，新认定省级以上企业技术中心1-2户，新产品投产50种以上。推进信息化带动工业化，加快无线城市建设。三是加快工业园区建设。坚持抓特色、强基础。全力加快镁工业园、硅藻土产业园、太阳能光伏产业园和长白山生态健康产业园等15个特色园区建设，主动对接国内外行业龙头、顶尖技术和知名品牌，引进节约型、环保型项目，延伸产业链，发展循环经济，形成产业集群和洼地效应。长白山天然矿泉水保护园区、八宝循环经济工业园、抚松人参产业园等园区争创省级特色工业园。四是全力抓好节能减排。坚持降能耗、促减排。全面开展年综合能耗3000吨标煤以上重点耗能企业节能监察工作，督促企业尽快淘汰落后产能、工艺和设备。二氧化硫、化学需氧量、氮氧化物、氨氮削减量完成省定指标，市中心区环境空气质量优良级别天数保持在325天以上，全市集中式饮用水源水质达标率100%。

　　(三)突出特色农业，致力于项目带动和产业化经营，着力加快新农村建设。坚持“重三化、强统筹，抓特色、上项目”，全力促进农业增效、农民增收和农村和谐。全市投放农业信贷资金 30亿元。农业总产值96.6亿元，增长15%。全力建设“六项”工程。

　　一是加快农业产业化工程建设。坚持“扶大、扶优、扶强”，加快17个3000万元以上产业化项目建设，年度投资8.3亿元，农业产业化产值达到 140亿元，增长20%。二是加快退粮进特工程建设。大力发展参、蛙、菌、药、菜、牧、果七大特色产业，引导农民退粮进特，建设60个1000亩以上退粮进特规模示范基地，全市完成退粮15万亩。特色产业乡镇达到20个、专业村100个、专业大户600个。三是加快人参产业工程建设。以提质增效、精深加工为重点，推进标准化生产基地、产业园区和质检平台建设，抓好省检中心和40个人参小区建设，人参GAP栽培面积稳定在2000万平方米，全市人参产业重点项目建设投资 10亿元以上，新产品开发上市20种以上，林下参面积增加到50万亩。加快日本津村、河北以岭药业、国药集团、山东绿叶、森工集团等合作项目建设，提高长白山人参精深加工能力和产业化水平。四是加快绿色产业工程建设。积极推进集体林权制度配套改革，全面落实经营权、处置权、收益权，促进林业快速发展。加快建设一批标准化绿色食品生产基地，带动农业向生态、优质、安全、高效方向发展。全市有机、绿色、无公害农产品认证数量达到370个、总产量160万吨。五是加快特色牧业振兴工程建设。重点支持特种野猪、中华蜜蜂、长白山梅花鹿、中国林蛙等种源繁育基地建设，全力打造吉林东部山区特色畜产品生产基地。完善质量安全监管体系，抓好重大动物疫病防控，确保重大疫病免疫率、屠宰检疫率100%。实现牧业产值18.5亿元，农村人均牧业收入 1200元。六是加快百村示范、村村提升工程建设。投资 8亿元以上，建设新农村项目450个，完成20个高标准示范村、20个整体推进村建设和100个村“四清四改”任务。

　　(四)突出商旅文化，致力于提档升级和基础建设，着力提升现代服务业水平。坚持龙头项目带动，全力促进以旅游为牵导的现代服务业提档升级。全年实现第三产业增加值 204亿元，增长24%。

　　一是重点发展旅游业。实施“双轮驱动，四季繁荣”战略，以长白山国际旅游度假区为龙头，推动旅游业转型升级，实现由单纯扩张规模向扩规模与提效益并重转变，由单一观光型向休闲养生度假复合型转变，由传统旅游方式向生态文化融合型转变。加快冰雪和温泉旅游基础设施建设，持续提升冰雪和温泉旅游对外影响力。加快旅游资源开发利用和区域整合，深度开发生态观光、休闲度假、康体养生、冰雪娱乐、民俗风情等特色旅游产品，积极推动域内外旅行社交流合作，打造长白山、鸭绿江精品旅游线路，开辟松花江水上黄金旅游线。加快推进仙人桥国际温泉城、龙山湖度假区、白山湖度假区建设。注重挖掘旅游文化内涵，高质量举办2012长白山鸭绿江文化旅游节等旅游文化节庆活动，塑造“神山圣水，天下白山”旅游目的地形象。加大松花石、黑陶、长白玉、紫砂系列等特色旅游商品开发力度。积极开展长白山国际旅游度假区创建国家5A级旅游景区活动，推动我市旅游产业快速发展。全年接待国内外游客478万人次，增长15%；旅游总收入50亿元，增长28%。二是优化升级商贸流通业。围绕繁荣城乡市场，调整优化商业网点布局，推进区域性中心市场和各类专业批发市场建设，提升商贸业的辐射力和拉动力。积极推进广泽城市综合体、欧亚商都、金英商业新区建设和长白山山货市场改造。加快再生资源回收加工利用产业园区建设，争取列入国家再生体系试点城市。加强农村流通体系建设，积极开展“金土地店店通”工程，推动农家店发展多种经营，提升综合服务功能。推进连锁经营，争取合兴大厦列入全省“农超对接”试点企业。确定 30户样本企业，建立市场监测信息平台。围绕构建区域物流中心，不断完善现代物流体系，加快靖宇丹东陆港建设，填补白山内陆港空白。积极谋划海铁联运、海陆联运、海关保税。三是大力发展文化产业。认真贯彻党的十七届六中全会精神，推进文化创新，丰富文化题材，挖掘传承发展长白山优秀传统文化，打造长白山文化品牌，努力促进文化产业大发展、大繁荣。加强文化市场体系建设，培育文化要素市场，不断完善文化流通网络，拓展大众文化消费市场。全力推进长白山民俗文化研究基地建设，推动博物馆、图书馆、艺术馆免费开放，大力扶持以长白山演艺公司、松花石文化产业园、抚松长白山文化创意产业园为代表的文化骨干企业。

　　(五)突出开发开放，致力于选优引强和扩量提质，着力增强经济发展活力。坚持“科学、理性、绿色、效益”招商理念，不断创新招商方式，招强选优、生态招商。全年引进市外资金 454亿元，增长25%；实际利用外资1.75亿美元，增长16.7%。

　　一是全力抓招商。强化产业招商，以硅藻土、矿泉水、人参、旅游等产业为重点，引进创新型、生态型、低碳型换代升级项目，每个优势产业谋划引进 10个 3000万元以上重点项目。强化定向招商，主要针对日韩在人参、绿色食品等产业，东南亚在旅游、医药等产业，欧美在矿产冶金、木制品、矿泉水等产业项目开展专题招商。境内主要以环渤海、长三角、珠三角地区为重点，巩固招商成果，拓宽合作领域。强化以商招商，积极参加东博会等经贸活动，办好第二届中国民营经济论坛，促成更多的项目和资本落户白山。强化责任招商，严格招商引资考核，建立完善项目跟踪督办机制，确保项目投资强度和建设周期。二是全力扩大开放。实施品牌培育工程，推动金隆木业等出口企业在国外商标注册、专利申请和质量认证工作。抢抓国家新一轮沿边开放政策，强化与产业互补城市、企业联姻，加强与图们江区域、东北东部地区合作，积极推动通钢矿业总部进驻白山，加快推进白通丹一体化和长白县国家级边境经济合作区进程，提升白山综合竞争力。全年实现外贸进出口总额 3.2亿美元，增长20%。

　　(六)突出开源节流，致力于税源建设和收支管理，着力壮大地方经济实力。坚持围绕效益抓经济，围绕财源上项目，不断壮大主导财源，培育新兴财源，储备后续财源，提高经济增长对财税贡献率。强化税收征管，搞好税源大普查，建立税源管控信息网络平台，加强煤炭、矿产、房地产等重点税种、重点行业、重点企业监管，严厉打击偷逃骗税行为，确保应收尽收。加强非税收入管理，增加政府可用财力。完善公共财政体制，调整优化支出结构，重点向教育、医疗卫生、就业和社会保障、社会救助、基础设施建设等方面倾斜。加强预算管理，大力压缩弹性支出，降低行政成本，厉行节约保重点、集中财力办实事。按照“产值分计、税收分享”的原则，积极构建市县(市)区经济一体化发展新格局。

　　(七)强化城乡统筹保障，致力于建管并举和营造精品，着力加快生态城镇化建设。坚持规划先行、建管并重、经营城市的理念，进一步提升城市品位、优化人居环境、打造精品亮点，建设更加舒朗大气、特色鲜明的山水园林城市，大幅提升城市综合竞争力。

　　一是加大城市建设力度。新建续建东岗街、白山南路、江源兴国路等 34条街路。完成市区污水处理主干网改造，改扩建垃圾处理场，实施“一江两岸”江堤亮化和公建、高层亮化工程。完成月牙河改造1300米、碱厂沟河征收拆迁和部分基础设施建设。加快福泰大厦、江源新城等旧城改造。规范房地产市场开发秩序，积极探索竞争开发和城市经营。二是加强城市管理。建立完善城市建设管理联席会议、重点工程会商审批、重点路段和小区清雪责任制、重点区域环卫包保和临街单位以及住户门前“五包”等相关制度，积极探索市区共建共管模式，下大气力整治重点路段占道经营，依法拆除违章建筑和规范广告牌匾。全面推进示范街路和示范小区创建工作，实施文明样板路工程，做到有序整洁。加强城市交通秩序整治，做到车辆按规定线路行驶，不乱停乱放。加强小区物业管理，保障水电气热供应，确保环境整洁、井然有序。三是加快交通建设。计划投资43亿元，建设里程 388公里。加快推进白山至长春、白山至临江 2条高速公路，加快松江河至长白高速前期工作。完善干线路网，改建临江大栗子至错草公路，提高省道石人至三道沟公路等级，改善白山至通化界路况，续建东岗至长白、三道沟至集安界边境通道。四是加速推进生态城镇化。全面推进浑江区、江源区一体化建设，做好重点连接城镇建设规划。加快抚松新城二期建设，计划征收安置居民 3000户，新建50万平方米安置房和商品房。推进靖(宇)松(赤松)一体化，加快建设现代化核电城和国际矿泉城，打造一批产业资本与生态资本互利共赢、人与自然和谐发展的宜居宜业精品城镇。

　　(八)强化社会民生保障，致力于协调发展和民生民利，着力建设和谐幸福白山。坚持科学发展，努力让城乡居民生活得更加美好，全力实施八大民生工程、办好32件民生实事，切实做到富民惠民安民。

　　一是暖房安居工程 5项：完成 123万平方米“暖房子”改造；完成江北北园小区、河口龙都小区、卫国小学东区城市棚户区和江源国有工矿棚户区改造；完成通沟、道清和南岭 D区煤矿棚户区改造；新建廉租房7.5万平方米；开工建设45.1万平方米沉陷区补充工程。二是社会保障工程 8项：全市养老保险、基本医疗保险、工伤保险、失业保险、生育保险参保人数分别达到 55.7万人、 96万人、22万人、12万人和21万人，覆盖率平均提高6个百分点以上；全市基本医疗保险限额由 4.5万元提高到5万元；城镇居民基本医疗统筹基金支付比例由60%提高到65%；力争实现城乡低保对象医疗救助“一站式”服务全覆盖，救助标准达到 2015元，增长16%；22672名完全并轨企业退休职工参加城镇职工基本医疗保险；启动实施城镇居民养老保险和第三批新农保试点工作；城市低保保障标准由月人均255元提高到280元、农村由年人均 1350元提高到1560元；建立全市城乡居民健康档案信息平台，电子档案建档率达到50%以上。三是社会进步工程8项：新建白山市逸夫特殊教育学校 200米标准跑道塑胶操场；扩建市体育馆健身房 1000平方米；力争每个县(市)区拥有1处标准放映场所；解决1000户偏远农村住户收听收看广播电视节目；完成殡仪馆一期建设；新建市级社会福利养老示范中心；完成市妇幼保健院、康宁医院主体工程建设；完成2900平方米救灾物资储备库建设。四是就业富民工程 5项：免费培训失业人员 1万人；新增发放特殊困难群体就业和创业小额贷款4500万元；城镇新增就业4万人，农村劳动力当年转移就业2.4万人，安排大学生见习人员350名，确保城镇零就业家庭动态为零；实施“强基富民固边”工程，努力解决边民社会保障、文化教育、医疗保障等问题；“兴边富民行动”争取补助资金2000万元，带动3000户农民致富。五是饮水安全工程 1项：新建农村安全饮水工程 130处，解决 7万人饮水安全问题。六是菜篮子工程 2项：在浑江两岸引水灌溉改造8000亩中低产田为无公害蔬菜基地；全市新增蔬菜大棚3000亩。七是便民出行工程2项：改造市区16万平方米人行步道；建设农村公路100公里。八是平安白山工程1项：推进“天网工程”，全市新增视频探头900个。

　　在做好民生实事的同时，大力实施质量兴市战略，积极培育一批拥有自主知识产权核心技术和市场竞争力强的特色产品，提升区域经济运行质量和效益。加强职业教育与特色产业智力支撑平台建设，推进县级职教中心实训基地建设，白山职业技术学校力争进入国家中等职业教育改革发展示范校和优质特色校行列。认真备战全国第十二届冬运会，力争夺取金牌。办好全国汽车场地越野锦标赛和全市第七届运动会。改造市体育场综合楼和新建冰雪训练基地。推进基层医疗卫生机构综合改革和公立医院改革试点。继续稳定低生育水平，人口自然增长率控制在 2.2‰以内。畅通维权投诉渠道，切实维护妇女儿童合法权益，为弱势群体提供社会救助。

　　(九)强化安全稳定保障，致力于加大整治和管理创新，着力构建平安白山。坚持稳定是第一责任，切实加强社会管理，确保群众安居乐业、社会安定有序，创造和谐稳定的社会环境，提高人民满意度。

　　一是切实抓好社会治安和安全生产工作。深入开展平安白山创建活动，保持高压严打态势，持续开展“打黑除恶”、社会治安整治等专项行动。深刻吸取血的教训，采取超常举措，做好煤矿资源整合，切实做到专人盯防，死看死守。加强非煤矿山、食品药品、道路交通、校安工程、公众聚集场所消防等公共安全专项整治，坚决遏制重特大事故发生，确保无重大森林火灾，保障人民群众生命财产安全。加强国防动员建设，进一步完善党政军警民合力治边的有效机制，深入开展“爱民固边暨平安边境模范市”创建活动。二是积极构建新型社会管理模式。进一步完善大案攻坚严打联动机制，强化网格化治安防控体系建设，建立社会公众广泛参与的邻里守望队伍。鼓励和支持社会各方更加积极有效地参与社会管理，善于运用群众路线的方式、民主的方式、服务的方式，积极化解人民内部矛盾。妥善处理群众来信来访，严格落实信访属地责任，强化信访考核约束力度，前移社会管理关口，力争个体访积案全部化解，当年案件结案率90%以上，重要时期进京非正常重复访实现动态为零。三是大力营造良好的法治环境。持之以恒地开展以精神文明建设为主要内容的各种创建活动，有针对性地开展“六五”普法教育，加大与经济发展、群众生产生活、社会和谐密切相关法律法规的宣传力度，巩固扩大成果，务求普法实效。弘扬知法守法、诚信文明的良好风尚，努力形成和谐社会人人有责、人人共享的生动局面。

　　三、履职尽责，奋发有为，切实加强政府自身建设

　　新的形势和任务，对政府工作提出了更高要求，我们要把“走基层、转作风、求实效、促发展”作为工作的总要求，抢抓机遇，奋勇争先，倾力打造务实、为民、高效、廉洁政府，始终对人民负责、受人民监督、让人民满意。

　　(一)善谋发展之策，激发政府创造力。不为定势所困，不为视野所限，不为艰难所惧，通过问计于基层、求教于群众、借智于专家，认真领会国家产业政策走向，强化优势资源、 10大产业调研，用足用好上级政策，找准结构调整与经济转型的突破口。加强学习培训，不断提升自身素质，努力超越自我，把先进发达地区的经验在白山嫁接提升，提高科学发展、统筹发展、快速发展、推动发展的本领，建设学习型政府，打造研究型队伍，使政府决策更具科学性、前瞻性、针对性和实效性。

　　(二)践行实干之风，提高政府执行力。全面推进政府工作提质提速，严格落实交办、催办、查办、督办制度，对年度各项目标任务进行细化分解，明确任务分工，明示流程时限，明晰责任奖惩。要着力转变文风会风，严格控制各类检查、评比和表彰，腾出更多的精力抓落实。要本着“资源信息共享、责任风险共担”的原则，进一步完善部门职能工作目标、机关建设共性目标和综合评议“三位一体”的绩效考核评估体系，理顺职责关系，坚持“一岗多责”，强化全方位行政问责和政务督查，营造奖勤罚懒、选优汰劣的良好发展氛围。

　　(三)履行依法之责，增强政府操作力。主动接受人大的法律监督和政协的民主监督，认真办理人大代表建议和政协委员提案，关注倾听各民主党派、工商联、无党派人士和人民团体意见，高度重视社会舆论和新闻媒体的监督。建立健全重大事项集体决策、专家咨询、社会公示、听证评估等制度，市政府常务会议邀请市人大、政协、军分区等相关领导参加。建立完善突发事件社会预警、应急救援、社会联动机制。扎实落实《行政强制法》，强化行政效能建设，推进政务中心标准化建设，清理规范行政许可审批事项，进一步优化发展软环境，全面提升科学理政、依法行政、从严执政的能力和水平。

　　(四)弘扬廉洁之气，提升政府公信力。建立完善政府投资项目廉政责任制，落实重大项目、重大决策、重点部门和大额资金使用保廉制度，按照项目建设“公开、公平、公正、公示”原则，实行部门审核、分管把关、会议讨论、集体决定，做到用制度管权、按制度办事、靠制度管人。强化对窗口单位、关键部门和实权岗位的重点监督，下大力气纠正部门和行业不正之风，努力解决行政缺位、越位、错位问题。坚持诚实守信，向群众社会承诺的事情做到“言必行、行必果”，树立清正廉洁、务实公信的政府整体新形象。

　　各位代表，新的形势催人奋进，发展使命任重道远。让我们在省委、省政府和市委的坚强领导下，开拓创新，奋力拼搏，全面完成明年各项目标任务，为实现白山在吉林东部率先崛起而努力奋斗！
